# In vivo cell proliferation analysis and cell-tracing reveal the global cellular dynamics of periodontal ligament cells under mechanical-loading

**DOI:** 10.1038/s41598-021-89156-w

**Published:** 2021-05-07

**Authors:** Masaru Mizukoshi, Masaru Kaku, Lay Thant, Kohei Kitami, Moe Arai, Isao Saito, Katsumi Uoshima

**Affiliations:** 1 Division of Orthodontics, Faculty of Dentistry and Graduate School of Medical and Dental Sciences, Niigata University, Niigata, Japan; 2 Division of Bio-Prosthodontics, Faculty of Dentistry and Graduate School of Medical and Dental Sciences, Niigata University, Niigata, Japan

**Keywords:** Stem-cell research, Regeneration, Multipotent stem cells

## Abstract

Periodontal ligament (PDL) is a uniquely differentiated tissue that anchors the tooth to the alveolar bone socket and plays key roles in oral function. PDL cells can respond rapidly to mechanical stimuli, resulting in accelerated tissue remodeling. Cell proliferation is an initial event in tissue remodeling and participates in maintaining the cell supply; therefore, analyzing cell-proliferative activity might provide a comprehensive view of cellular dynamics at the tissue level. In this study, we investigated proliferating cells in mouse molar PDL during orthodontic tooth movement (OTM)-induced tissue remodeling. Our results demonstrated that the mechanical stimuli evoked a dynamic change in the proliferative-cell profile at the entire PDL. Additionally, cell-tracing analysis revealed that the proliferated cells underwent further division and subsequently contributed to tissue remodeling. Moreover, OTM-induced proliferating cells expressed various molecular markers that most likely arise from a wide range of cell types, indicating the lineage plasticity of PDL cells in vivo. Although further studies are required, these findings partially elucidated the global views of the cell trajectory in mouse molar PDL under mechanical-loading conditions, which is vital for understanding the cellular dynamics of the PDL and beneficial for dental treatment in humans.

## Introduction

Periodontal ligament (PDL) is a uniquely differentiated tissue existing between two mineralized tissues (root surface cementum and alveolar bone), with the thin, non-mineralized layer maintained under the physiological state. PDL plays critical roles in oral function, including tooth support, dissipation of masticatory forces, neurological feedback, regulation of tooth eruption, and orthodontic tooth movement (OTM)^1^. Owing to the multi-layered structure, PDL harbors various cell types, including fibroblasts, endothelial cells, osteoblasts, cementoblasts, and osteoclasts^2^. To maintain such a variety of cell types, PDL is considered abundant in stem cells. In fact, PDL-derived cells possess high proliferation and multi-differentiation potential under culture^3^. Although the cell characteristics of PDL stem cells in vitro are relatively well-studied^4^, those in vivo remain largely unknown. Elucidating the tissue localization of the stem cells and their differentiation kinetics in vivo would enhance our understanding of the homeostatic mechanism of the PDL.

Previous studies show that mesenchymal stem cell (MSC)-marker (e.g., STRO-1, CD146, CD44)-positive cells are distributed in the perivascular region of the PDL^5,6^. Because the bone side of PDL is rich in vasculature, MSCs are abundant at the bone side of PDL relative to the cementum side^7^. Recent studies suggest that specific cell populations contribute to the tissue maintenance of PDL. Hedgehog-signaling-responsive Gli1-lineage cells are present at the apical region and spread to the entire PDL in response to injury and OTM^8,9^. Moreover, α-smooth muscle actin^10^, Osterix/Sp7^11^, parathyroid hormone-related peptide^12^, Axin2^13^, and CD90/Thy1^14^-lineage cells also expand in the PDL space, indicating that these cells play possible roles in PDL tissue maintenance. Although each cell type contributes to the tissue maintenance of PDL, they have a distinct distribution pattern and do not comprise the entire PDL cell population. Thus, further studies are required to understand the global behaviors of PDL cells at the tissue level.

In the continuously growing mouse incisor, the entire spectrum of developmental and adult stages of the cells is seen throughout life; thus, it is widely used to study the differentiation trajectory of stem cells. In contrast to the highly proliferative nature of stem cells under culture, stem cells remain quiescent in vivo and reside in a specialized microenvironment. In the mouse incisor, a small population of slow-cycling MSCs exists at the proximal end of the epithelial cervical loops^15^. Immediately distal to the stem cell compartment, rapidly cycling, undifferentiated, transit-amplifying cells (TACs) are present^16^. Rapid-cycling TACs in mouse incisor mesenchyme express specific genes, such as polycomb repressive complex 1 (PRC1)^17^. Targeted deletion of PRC1 components, such as Ring1a and Ring1b, leads to the loss of TACs and results in the arrest of incisor growth^16^. This suggests that the transition of slow-cycling MSCs to rapidly cycling progenitor cells is a critically important process for tissue maintenance. Therefore, analyzing the cell-proliferative activity might offer a comprehensive view of the cell-differentiation trajectory at the tissue level.

Although cell proliferation is a favorable indicator of cell-differentiation trajectory, mouse molar PDL, as well as human tooth PDL, are stationary in terms of proliferative activity under physiological conditions. A unique feature of PDL cells is a rapid response to mechanical stimuli, which is well documented during OTM^8,18^. Therefore, we conceived the idea that changes in mechanical-loading-induced cell proliferation activity would expose the cell-differentiation trajectory in mouse molar PDL. Here, we determined the detailed characteristics of mechanical-loading-induced proliferating cells and the fate of these cells in order to obtain a comprehensive view of the cellular dynamics in PDL.

## Results

### Cell-proliferative status during OTM

**Figure 1:**
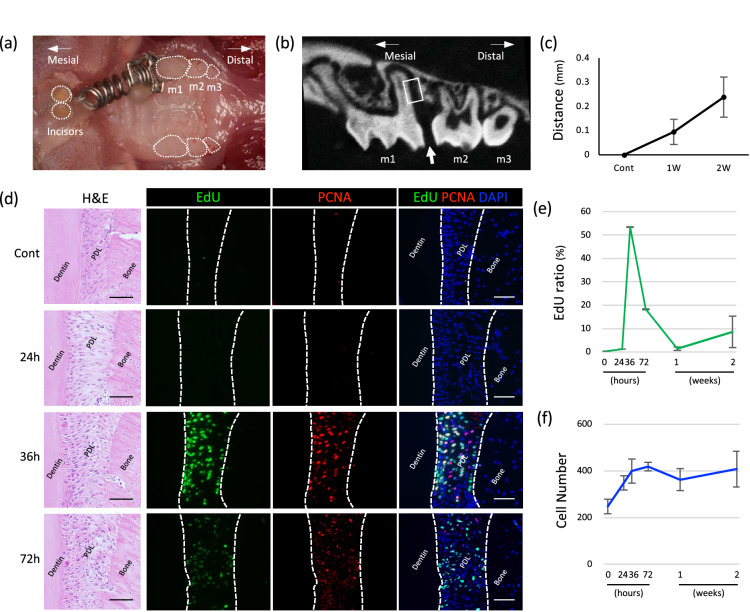
Distribution of proliferating cells in the PDL during OTM. (**a**) OTM of mouse molar. An Ni–Ti coil was placed between the maxillary bilateral incisors and the left first molar of 8-week-old male mice. (**b**) Micro-CT image of maxilla at 2 weeks of OTM. Widening of the distance between the first and second molar was evident (arrow). Distal side PDL of the distal root was chosen as the region of interest (white rectangle). (**c**) Distance between the first and second molars increased over time. (**d**) H&E staining of the histology sample from the distal side PDL of the distal root. Extension of the PDL fibers was evident after 24 h and persisted for up to 72 h. Both EdU^+^ cells (green) and PCNA^+^ cells (red) appeared at 36 h of OTM. The proliferating cells were distributed evenly in the PDL. Bar: 50 μm. (**e**) The EdU^+^ cells exponentially increased at 36 h of OTM and decreased as time proceeded. (**f**) The total number of cells in the PDL increased mainly at the early stage of OTM.

The proliferative status of the mouse molar PDL is stationary under physiological conditions, similar to human tooth PDL. To expose the proliferative activity of cells in mouse molar PDL, we used an OTM model, in which tissue remodeling is accelerated by constant mechanical-loading using a coil spring (Fig. 1a). In the control, the distance between the first and second molars was 0.0 ± 0.0 μm. At 1 and 2 weeks, the distance increased to 95.0 ± 52.0 μm and 238.6 ± 83.0 μm (n = 5), respectively (Fig. 1b,c), confirming that the first molar was moved to the mesial side, resulting in the mesial side PDL receiving compressive force, and the distal PDL suffering tension force. We chose the distal side PDL of the distal root (i.e., the side with tension) as the region of interest (Fig. 1c, white rectangle). Hematoxylin and eosin (H&E) staining showed that extension of the PDL fibers was evident at as early as 24 h of OTM. (Fig. 1d), with extension and dis-alignment of the PDL fiber persisting for up to 72 h. Proliferating cells among PDL cells were detected using 5-ethynyl-2′-deoxyuridine (EdU) incorporation and the PCNA antibody. EdU^+^ cells were rarely detected in the control, but they exponentially increased at 36 h of OTM and then decreased as time progressed (n = 3) (Fig. 1e). The PCNA^+^ cells showed the same tendency but did not fully overlap with the EdU^+^ cells shown in the superimposed image at 36 h. The proliferating cells were distributed evenly in the PDL, and we observed no correlation with the architectural component, such as blood vessels and mineralized tissue boundaries. Moreover, following the early response of the proliferating cells to the OTM, the total cell number increased in PDL (n = 3) (Fig. 1f).

**Figure 2:**
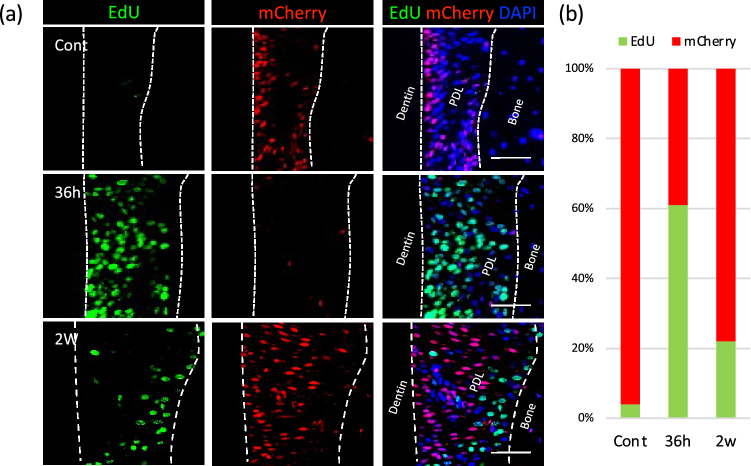
Cell cycle status of PDL cells during OTM. (**a**) Changes in cell cycle status were visualized using Fucci2 mice injected with EdU. The majority of the PDL cells expressed mCherry (red) in control, indicating their status in the quiescent phase. At 36 h, the quiescent cells (red) decreased dramatically, corresponding to increases in proliferating cells (green). At 2 weeks, the majority of the cells returned to the quiescent phase while proliferative activity persisted in some cells. Bar: 50 μm. (**b**) Cell cycle profile showing temporal changes in PDL cell-proliferative activity during OTM.

We then analyzed the cell-proliferative status using a Fluorescent Ubiquitination-based Cell Cycle Indicator (Fucci2) mouse^19,20^. In this mouse, the cells at the S/G2/M phase express mVenus (green fluorescence protein) under control of the hGem (1/110) promoter, and cells in the G1 phase express mCherry (red fluorescence protein) under control of the hCdt1(30/120) promoter, thereby allowing cell cycle status to be distinguished by two different fluorescence probes. However, the mVenus signal was not detected in the PDL of 8-week-old mice (Supp. Fig. 1). Therefore, we used EdU administration to label proliferating cells in Fucci2 mice. In the control, the majority of PDL cells expressed mCherry, indicating that they were in the quiescent phase (Fig. 2a). At 36 h, the quiescent cells decreased dramatically, corresponding to an increase in the number of proliferating cells. At 2 weeks, the majority of cells returned to the quiescent phase, whereas proliferative activity persisted in some cells (n = 3) (Fig. 2b).

### Characterization of the proliferating cells during OTM

**Figure 3:**
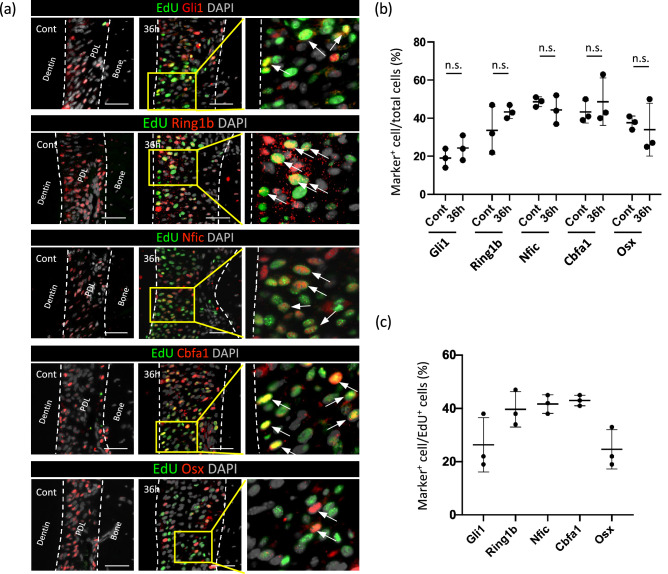
Characterization of proliferative cells during OTM. (**a**) Marker expression (red) in EdU^+^ proliferating cells (green) after 36 h of OTM. The endogenous expression of each marker is shown in the control. Marker^+^ cells among proliferating cells are indicated by arrows. Bar: 50 μm. (**b**) Ratio of marker^+^ cells among total PDL cells between control and at 36 h of OTM were compared. OTM did not significantly affect the expression of the markers analyzed. (**c**) Percentage of marker^+^ cells among the EdU^+^ proliferating cells at 36 h of OTM. Proliferating cells expressed a diverse range of markers.

To further characterize the OTM-induced proliferating cells in PDL, we analyzed the expression of cellular markers by immunohistochemistry (Fig. 3). We chose the following markers: Gli1 for MSCs; Ring1b for TACs; and Nfic, Cbfa1, and Osx for root/PDL-forming cells. Representative images of the immunoreactivity to the respective antibodies (red) together with the proliferating cells (green) at 36 h of OTM are shown in Fig. 3a. At 36 h of OTM, the ratio of marker^+^ cells among total PDL cells was analyzed, and no significant difference was observed compared to control (n = 3) (Fig. 3b). Additionally, the ratio of marker^+^ cells within the EdU^+^ proliferating cells showed a diverse expression pattern of the markers, indicating that the proliferating cells comprised a wide range of cell types (n = 3) (Fig. 3c).

### Fate of OTM-induced proliferating cells

**Figure 4:**
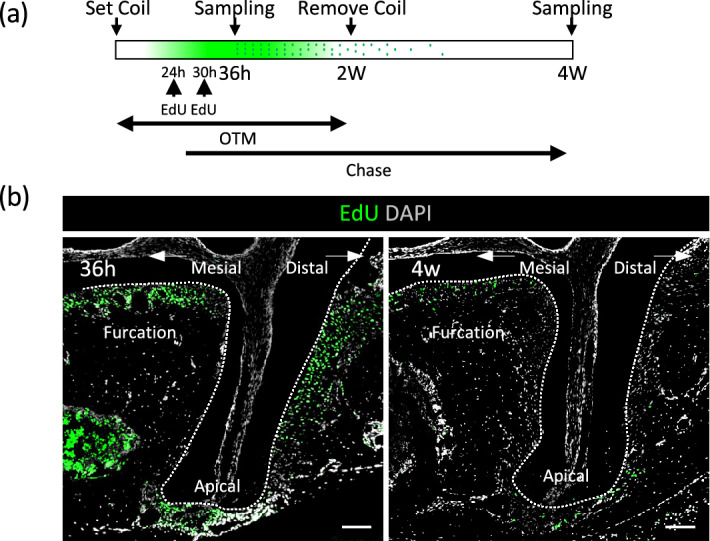
Fate of OTM-induced proliferating cells. (**a**) Schematic experimental schedule of proliferated-cell-tracing. EdU-labeled cells at 36 h after OTM were further traced for 4 weeks. (**b**) Proliferative cells were detected at the distal side of the distal root, apical, and furcation areas of the PDL (36 h). After 4 weeks of chasing, EdU-labeled cells disappeared completely at the distal side PDL of the distal root, whereas some EdU-labeled cells remained present at the apical and furcation areas of the PDL (4 weeks). Bar: 200 μm.

EdU-labeled cells at 36 h after OTM were further traced for 4 weeks (Fig. 4a). Proliferative cells were detected at the distal side of the distal root, apical, and furcation areas of the PDL at 36 h after OTM (Fig. 4b). After 4 weeks of chasing, the EdU-labeled cells completely disappeared at the distal side PDL of the distal root, whereas EdU-labeled cells remained present at the apical and furcation areas of the PDL.

**Figure 5:**
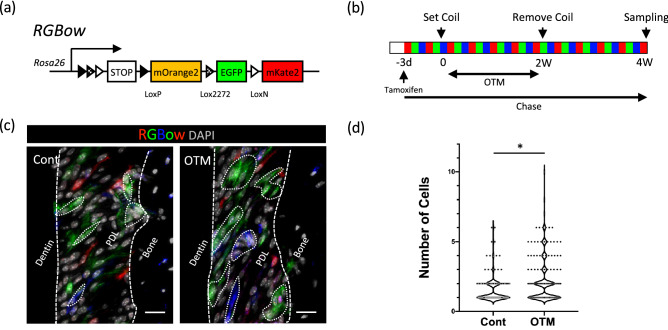
In vivo cell clonal analysis. (**a**) Schematic of the RGBow reporter construct. Tamoxifen-dependent Cre-recombinase generates three fluorescence proteins at random. (**b**) Schematic experimental schedule of in vivo cell clonal analysis. (**c**) Representative images of the analysis using RGBow: UBC-CreER^T2^ mice. Single-cell-derived cell clones were visualized with three pseudo colors (red, green, or blue). The cell clusters are marked with a dotted line. Bar: 50 μm. (**d**) The number of cells in each cluster increased significantly from 1.5 ± 0.9 to 2.2 ± 1.6 by OTM. *p < 0.05.

In vivo cell clonal analysis was performed using RGBow: UBC-CreER^T2^ (RGBow) mice, where all cells randomly expressed three different fluorescence probes in a tamoxifen-inducible manner (Fig. 5a, b). OTM was initiated in 8-week-old RGBow mice, and tamoxifen was injected 3 days prior to the OTM. After 2 weeks of OTM and an additional 2 weeks of tracing, cluster formation was analyzed histologically (Fig. 5c). Single-cell-derived cell clones were visualized with three pseudo colors (red, green, or blue). The number of cells in each cluster increased significantly from 1.5 ± 0.9 to 2.2 ± 1.6 by OTM, confirming further division of the OTM-induced proliferating cells (n = 3) (Fig. 5d). Consistent with the distribution of proliferating cells, we observed a non-specific distribution of the single-cell-derived clusters within PDL. Notably, the cell clusters tended to grow along the collagen fiber direction of the PDL.

## Discussion

Cell proliferation is an initial event of tissue remodeling and plays a key role in maintaining the cell supply. Although accelerated cell proliferation under mechanical-loading conditions has been reported previously^18,21^, it remains unclear which cells acquire proliferative ability and from where these cells arise. Here, we showed that proliferating cells exponentially increased at 36 h after OTM. OTM-induced proliferating cells appeared at the entire PDL and did not have a geological correlation to the vasculature, a putative stem cell location. Moreover, single-cell-derived clusters in RGBow mice, representing daughter cells of the proliferated cells, did not exhibit a specific distribution pattern. Changes in cell cycle status in response to OTM were also detected at the entire PDL, as confirmed in Fucci2 mice. Furthermore, OTM-induced proliferating cells expressed the TAC marker Ring1b, as well as various other molecular markers, including Gli1, Nfic, Cbfa1, and Osx. These results, including the non-preferential distribution and expression of a wide range of molecular markers, indicate that OTM-induced proliferating cells do not arise from specific cell types and locations.

Although Ring1b expression is strictly restricted to TACs in mouse incisor mesenchyme, it is widely distributed in the mouse molar PDL, and the ratio of Ring1b-expressing cells was unaltered in response to OTM. These results indicate that Ring1b-expressing proliferating cells are not representing TACs in mouse molar PDL. Furthermore, it is likely that the differentiation trajectory mechanism represented by the dynamic change in cell-proliferative activity (i.e., the transition from slow-cycling MSCs to rapid-cycling TACs) does not exist in mouse molar PDL under mechanical-loading conditions.

Nfic is a member of the nuclear factor I family and functions as a critical regulator of root formation, with this supported by defective root formation observed in *Nfic*-knockout mice while crown formation remains intact^22^. Nfic interacts with TGF-β signaling and is also a downstream target of Gli1-mediated hedgehog signaling^23^. Additionally, Nfic reportedly promotes the proliferation and osteogenic/cementogenic differentiation of dental follicle cells^24^. In the present study, we identified Nfic expression in ~ 50% of the cells in mouse molar PDL, as well as some of the proliferating cells. According to previous reports, Nfic might also play roles in PDL homeostasis, as seen during tooth root/PDL development^22,23^.

Although the number of Gli1- and Cbfa1-expressing cells reportedly increases at 7 days^8^ and Osx-expressing cells at 48 h of OTM^25^, we did not observe any changes in these markers at 36 h of OTM. In general, terminal differentiation usually coincides with proliferation arrest; thus, regulation of cell cycle and cell-type-specific transcription has a unique inverse relationship^26^. In accordance with this concept, we observed the appearance of proliferating cells and the disappearance of quiescent cells concurrently in Fucci2 mice. Furthermore, these data highlight the involvement of temporal coordination between cell cycle and terminal differentiation in PDL cells.

In the present study, we selected the distal PDL of the distal root as ROI since mechanical-loading-induced cell proliferation was consistently evoked in this region. Besides, this region has a typical ligament structure (i.e., rich in oriented collagen fibers and less vasculature) and load-bearing environment. However, recent studies indicated that the PDL is not uniform; different regions have distinct molecular and mechanical characteristics^27,28^. For instance, the PDL at the furcation area is sparse in collagen fiber, resulting in lower stiffness and a distinct protein composition compared to the collar region^27^. Moreover, the force distribution of the furcation area is different, as confirmed by finite element analysis^29^. Since the extracellular environment crucially affects cell behaviors, different regions of PDL may have different cell-differentiation trajectories.

To analyze the fate of OTM-induced proliferating cells in PDL, EdU-labeled proliferated cells were further traced for 4 weeks. The EdU-labeled cells completely disappeared at the distal PDL of the distal root, indicating that the cell division was not transient, and that these cells undergo further division and contribute to tissue remodeling. Cluster formation of single-cell-derived cells in RGBow mice also supported this observation. Remnants of the EdU-labeled cells at the apical and furcation area suggested that these cells did not divide further, most likely due to the distinct molecular and mechanical environment in this region, as discussed earlier.

It has long been believed that a small number of tissue stem cells are responsible for tissue remodeling. Although we detected a dramatic change in the proliferative status in response to mechanical-loading, the transition of slow-cycling MSCs to rapidly cycling TACs confirmed in mouse incisor mesenchyme^17^ was not evident in mouse molar PDL during OTM. Conversely, the proliferative cells arise from various cell types at entire PDL tissue, this may suggest the differentiation plasticity of the PDL cells. A recent study showed that distraction osteogenesis induces the focal adhesion kinase signaling pathway and activates a gene-regulatory program usually activated in primitive neural crest cells, which is a major stem cell population during craniofacial development^30^. Similar to distraction osteogenesis, OTM represents mechanical-loading-induced tissue remodeling and PDL cells also originate from the neural crest^31^, the proliferative cells during OTM might be similar to the primitive neural crest cell and capable of giving rise to multiple dental mesenchymal cells. Although PDL cells act as fibroblasts to maintain collagen-rich fibrous tissue at the physiological state^32^, they might also exhibit lineage plasticity according to feedback signals from the surrounding environment^33^. In line with this notion, the multi-differentiation potential of PDL-derived cells into non-dental cells, including chondrocytes^34^, cardiac myocytes^35^, pancreatic-islet cells^36^, and retinal ganglion-like cells^37^, has been well documented in culture conditions.

In summary, we analyzed proliferating cells in mouse molar PDL during mechanical-loading-induced tissue remodeling. The results demonstrated that the mechanical stimuli evoked a dynamic change in the cell-proliferative profile at the entire PDL, and the proliferated cells contributed to tissue remodeling. Additionally, these findings indicated the lineage plasticity of PDL cells in vivo. Although further studies are required, our findings partially elucidate the global views of the cell-differentiation trajectory in mouse molar PDL under mechanical-loading conditions. Understanding the cellular dynamics is vital to maintain tissue homeostasis in a clinical setting and to develop innovative regenerative strategies in humans.

## Methods

### Ethics statement

The use of animals and all animal procedures in this study were approved by the Niigata University Animal Experiment Ethics Committee (approved protocol number: SA00067), and performed in accordance with the Guidelines for Proper Conduct of Animal Experiments specified by the Science Council of Japan. All animal handling and experiments were in compliance with the ARRIVE Guidelines for animal research reporting of in vivo experiments.

### Animals

Wild-type mice (C57BL/6J, male) were purchased from Charles River Laboratories Japan (Yokohama, Japan). An R26p-Fucci2 mouse (CDB0203T)^19,20^ was obtained from the RIKEN Center for Life Science Technologies. A UBC-CreER^T2^ mouse (JAX007001)^38^ and RGBow mouse (JAX028583)^39^ were obtained from Jackson Laboratory (Bar Harbor, ME, USA). All mice were maintained in the animal facility of Niigata University.

### OTM

The Ni–Ti coil (25 gf; TOMY, Tokyo, Japan) was placed between the maxillary bilateral incisors and the left first molar of 8-week-old male mice with stainless steel wire (φ 0.09 mm). The steel wire tied to the incisors was reinforced with a light cure composite resin (BEAUTIFIL Flow; Shofu, Kyoto, Japan) to prevent breakage during mastication. After placing the Ni–Ti coil for a defined period of time (24, 36, and 72 h; 1 and 2 weeks), both sides of the maxilla were harvested and fixed with 10% paraformaldehyde for 24 h. The upper right first molar served as a control.

### Nucleoside incorporation for proliferating cells

EdU (Invitrogen, Carlsbad, CA, USA) was intraperitoneally injected (5 mg/100 g body weight) into the wild-type or Fucci2 mice at 6 h and 12 h prior to euthanasia.

### Micro-CT

Micro-CT images of the maxilla were obtained using 90-kV and 88-μA irradiation from a high-resolution X-ray tomographic system (CosmoScan GX; Rigaku Corporation, Tokyo, Japan). Structural analysis was performed using a software tool (Analyze 12.0; Analyze Direct, Inc., Overland Park, KS, USA). The outline of the teeth was defined with a threshold intensity between 6,000 and 12,000 Hounsfield units. The distance between the closest point of the first and second molars was measured.

### Histology and immunohistochemistry

To prepare the paraffin-embedded histology sections, samples were decalcified with 10% EDTA for 3 weeks at room temperature after image collection by micro-CT. Sections were stained with H&E for morphological analysis.

EdU-incorporated proliferative cells were detected using the Click-iT EdU Cell Proliferation Kit for Imaging Alexa Fluor 488 dye (Invitrogen) according to manufacturer instructions. Following the detection of EdU-labeled cells, molecular markers were co-labeled by immunohistochemistry, as previously described^40^. The primary antibodies used were as follows: mCherry (1:2,000; M11217; Invitrogen), Gli1 (1:400; 78259; NOVUS, Littleton, CO, USA), Ring1b (1:100; 101273; Abcam, Cambridge, UK), Cbfa1/Runx2 (1:400; 035; MBL Life Sciences, Nagoya, Japan), Sp7/Osterix (1:800; 22552; Abcam), and PCNA (1:200; 2586; Cell Signaling Technology, Danvers, MA, USA). Secondary antibodies were as follows: goat anti-rabbit IgG antibody Alexa Fluor 555 (1:400; Invitrogen) and goat anti-mouse IgG antibody Alexa Fluor 555 (1:400; Invitrogen). For the detection of Gli1, Alexa Fluor 555 Tyramide SuperBoost Kit goat anti-rabbit IgG (Invitrogen) was used to amplify the immunoreactivity according to manufacturer instructions. The samples were enclosed with VECTASHIELD Vibrance Antifade Mounting Medium with DAPI (Vector Laboratories, Inc., Burlingame, CA, USA).

### Cell clonal analysis

Three days before applying the coil, tamoxifen (T5648; Sigma-Aldrich, St. Louis, MO, USA) was injected intraperitoneally (75 mg/kg body weight) into RGBow: UBC-CreER^T2^ mice. After 14 days of OTM, the coil was removed, and mice were sacrificed after an additional 14 days of tracing. Paraffin-embedded histology sections were prepared, as described, and fluorescence proteins (EGFP; mOrange2 and mKate2) were detected with the following primary antibodies: EGFP (chicken anti-GFP antibody: 1:2,000; 13970; Abcam), mOrange2 (rat anti-mCherry antibody: 1:200; M11217; Invitrogen), and mKate2 (rabbit anti-tRFP antibody; 1:200; AB233; Evrogen, Moscow, Russia). The secondary antibodies used were as follows: goat anti-rat IgG antibody Alexa Fluor 488 (1:400; Invitrogen), goat anti-chicken IgY antibody Alexa Fluor 555 (1:400; Invitrogen), and goat anti-rabbit IgG antibody Alexa Fluor 633 (1:400; Invitrogen).

### Image capturing

A system microscope (BX53; Olympus, Tokyo, Japan) equipped with a digital camera (DP80; Olympus) was used to capture images. Fiji/ImageJ (NIH, Bethesda, MD, USA) and cellSens (Olympus) were used for image processing.

### Statistical analysis

The distal side of the PDL on the distal root of the first maxillary molar was selected as the observation area. The number of EdU^+^ cells, immuno-positive cells, and total cells within the observation area were counted, and the positive cell ratio was calculated. Cell counting was performed on at least three different samples. For statistical analyses, an unpaired *t*-test with Welch’s correction was performed using GraphPad Prism (GraphPad Software, San Diego, CA, USA), and a p < 0.05 was considered significant.

## Data availability

The data that support the findings of this study are available within the article. Additional data may be available from the corresponding author, [M.K.], upon reasonable request.

## Supplementary Information

Supplementary Information.
