# Supplementary material for: In vivo cell proliferation analysis and cell-tracing reveal the global cellular dynamics of periodontal ligament cells under mechanical-loading
Source: Sci Rep. 2021 May 7;11:9813. doi: 10.1038/s41598-021-89156-w (PMC8105403; doi:10.1038/s41598-021-89156-w)
Supplement: Supplementary file 1 — Supplementary Information. [file 41598_2021_89156_MOESM1_ESM.pdf]

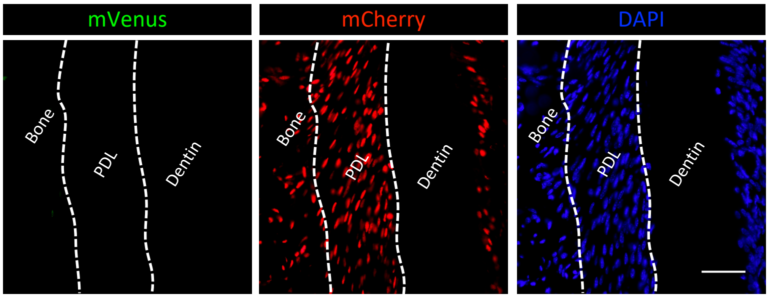


**Supp. Fig. 1. Detection of fluorescent signals on PDL tissue of Fucci2 mouse.** Fluorescence signals (mVenus for proliferating cells and mCherry for resting cells) on PDL tissue of Fucci2 mouse (8-week-old) were analyzed by immunohistochemistry. The fluorescence signal of mVenus was not detected, while that of mCherry was clearly detected. We tested three different anti-GFP polyclonal antibodies (A11122; Invitrogen. Ab13970; Abcam, 598; MBL), which are confirmed to detect mVenus, but none could not detect the mVenus on the PDL tissue of Fucci2 mouse. The original reference for the Fucci2 mouse also reported that the mVenus signal was not detected in adult tissues except testis[19]. Bar: 50 µm.
